# Physical Exercise and Alzheimer’s Disease: Effects on Pathophysiological Molecular Pathways of the Disease

**DOI:** 10.3390/ijms22062897

**Published:** 2021-03-12

**Authors:** Susana López-Ortiz, Jose Pinto-Fraga, Pedro L. Valenzuela, Juan Martín-Hernández, María M. Seisdedos, Oscar García-López, Nicola Toschi, Francesca Di Giuliano, Francesco Garaci, Nicola Biagio Mercuri, Robert Nisticò, Enzo Emanuele, Simone Lista, Alejandro Lucia, Alejandro Santos-Lozano

**Affiliations:** 1 i+HeALTH Research Group, Department of Health Sciences, European University Miguel de Cervantes, 47012 Valladolid, Spain; slopezo@uemc.es (S.L.-O.); fjpinto@uemc.es (J.P.-F.); jmartinh@uemc.es (J.M.-H.); maria.medina.seisdedos@gmail.com (M.M.S.); asantos@uemc.es (A.S.-L.); 2 Faculty of Sport Sciences, Universidad Europea de Madrid, Villaviciosa de Odón, 28670 Madrid, Spain; pedroluis.valenzuela@universidadeuropea.es (P.L.V.); oscar.garcia@universidadeuropea.es (O.G.-L.); simone.lista@icm-institute.org (S.L.); 3 Department of Biomedicine and Prevention, University of Rome “Tor Vergata”, 00133 Rome, Italy; toschi@med.uniroma2.it (N.T.); francesco.garaci@uniroma2.it (F.G.); 4 Department of Radiology, “Athinoula A. Martinos” Center for Biomedical Imaging, Boston, MA 02129, USA; 5 Harvard Medical School, Boston, MA 02115, USA; 6 Neuroradiology Unit, Department of Biomedicine and Prevention, University of Rome “Tor Vergata”, 00133 Rome, Italy; francescadigiuliano@msn.com; 7 Casa di Cura “San Raffaele Cassino”, 03043 Cassino, Italy; 8 Department of Experimental Neuroscience, IRCCS Fondazione Santa Lucia, 00143 Rome, Italy; mercurin@med.uniroma2.it; 9 Department of Systems Medicine, University of Rome “Tor Vergata”, 00133 Rome, Italy; 10 Laboratory of Pharmacology of Synaptic Plasticity, EBRI Rita Levi-Montalcini Foundation, 00161 Rome, Italy; robert.nistico@gmail.com; 11 School of Pharmacy, University of Rome “Tor Vergata”, 00133 Rome, Italy; 12 2E Science, Robbio, 27038 Pavia, Italy; enzo.emanuele@2escience.com; 13 Research Institute of the Hospital 12 de Octubre (“imas12”), 28041 Madrid, Spain; 14 Centro de Investigación Biomeédica en Red Fragilidad y Envejecimiento Saludable (CIBERFES), 28029 Madrid, Spain

**Keywords:** physical exercise, Alzheimer’s disease, amyloid-β peptide, tau protein, molecular pathways

## Abstract

Alzheimer’s disease (AD), the most common form of neurodegenerative dementia in adults worldwide, is a multifactorial and heterogeneous disorder characterized by the interaction of genetic and epigenetic factors and the dysregulation of numerous intracellular signaling and cellular/molecular pathways. The introduction of the systems biology framework is revolutionizing the study of complex diseases by allowing the identification and integration of cellular/molecular pathways and networks of interaction. Here, we reviewed the relationship between physical activity and the next pathophysiological processes involved in the risk of developing AD, based on some crucial molecular pathways and biological process dysregulated in AD: (1) Immune system and inflammation; (2) Endothelial function and cerebrovascular insufficiency; (3) Apoptosis and cell death; (4) Intercellular communication; (5) Metabolism, oxidative stress and neurotoxicity; (6) DNA damage and repair; (7) Cytoskeleton and membrane proteins; (8) Synaptic plasticity. Moreover, we highlighted the increasingly relevant role played by advanced neuroimaging technologies, including structural/functional magnetic resonance imaging, diffusion tensor imaging, and arterial spin labelling, in exploring the link between AD and physical exercise. Regular physical exercise seems to have a protective effect against AD by inhibiting different pathophysiological molecular pathways implicated in AD.

## 1. Introduction: Physical Activity and Alzheimer’s Disease

Life expectancy is increasing steadily worldwide [1], and this population trend has been associated with an increase in the incidence of chronic diseases [2,3]. For instance, neurodegenerative diseases are one of the major causes of morbidity and mortality in older individuals, and many studies agree that dementia, particularly, Alzheimer’s disease (AD) is among the most prevalent conditions in this age group [4,5,6]. Indeed, AD and other dementias were the 7th leading cause of death worldwide in 2019 [7].

AD, a chronic and progressive neurodegenerative disease characterized by insidious cognitive deterioration [8], shows a high degree of pathophysiological complexity and clinical heterogeneity [9,10]. The extracellular deposition of accumulated amyloid beta (Aβ) peptide (i.e., the 42 amino acid-long Aβ peptide [Aβ_1–42_]) in the form of diffuse and neuritic Aβ plaques and the intraneuronal accumulation of neurofibrillary tangles (NFTs), consisting of aggregated hyperphosphorylated tau (p-tau) proteins, are the key pathophysiological hallmarks detected in AD brains [11]. These features are accompanied by loss of synapses and selective neuronal cell death [12].

Aging is the most relevant risk factor for sporadic AD. In this regard, in the U.S., the prevalence of AD is around 10% in individuals over 65 years of age, around 32% over 85 years, and around 50% over 95 years [13]. In AD brains, neurons show aging hallmarks, such as destabilization of the genome molecular integrity, telomere length reduction, modified epigenetic signatures, and mitochondrial dysfunction [14]. Notably, in AD, a significant imbalance between Aβ production and clearance molecular mechanisms has been reported as a primary cause of Aβ dyshomeostasis along with protein misfolding, aggregation, and consequent extracellular accumulation of Aβ plaques [15]. In early-onset AD (EOAD), this imbalance is mainly the result of a genetic-driven deregulation of the amyloidogenic pathway leading to excessive production of Aβ [16]; in late-onset AD (LOAD), an overall impairment of brain Aβ clearance is assumed to arise [17].

Genetic susceptibility is a key factor in determining and affecting AD onset and pathogenesis. EOAD forms (age at onset younger than 65 years) include 5% to 6% of all AD cases, encompass a significant percentage of phenotypic variants differing from the amnestic presentation of typical AD, and show a marked genetic predisposition [18]. Particularly, three EOAD genes—amyloid precursor protein (*APP*) [19], presenilins 1 and 2 (*PSEN1* [20] and *PSEN2* [21,22])—show high-penetrant mutations inherited as autosomal dominant trait [23,24,25] (i.e., autosomal dominant AD [ADAD] forms representing <1% of all AD cases) [26,27]. Currently, over 300 autosomal dominant mutations have been documented across *APP*, *PSEN1*, and *PSEN2* genes) [28]. However, based on the investigations performed so far, the majority of EOAD patients remain genetically unexplained (i.e., non-familial or sporadic EOAD forms) [18,27]. The missing genetic etiology of EOAD cases is further complicated by their high phenotypic heterogeneity.

LOAD forms (age of onset of 65 years or older) are polygenic and show multifactorial causes. Notably, polymorphism in the apolipoprotein E (*APOE*; locus on chromosome 19q13.2) gene is a crucial genetic risk determinant of LOAD, with the *APOE ε4* allele conferring an increased risk and the *APOE ε2* allele conferring a decreased risk relative to the common *APOE ε3* allele [29]. Several Aβ-dependent and Aβ-independent pathways are differentially regulated by APOE isoforms. In addition, more than 50 genes/loci associated with the risk of developing LOAD have been disclosed so far via large-scale genomics studies, including genome-wide association studies (GWAS), meta-analyses of GWAS, and next-generation sequencing (NGS)-based analyses [30,31,32]. These approaches facilitated the investigation of disease pathomechanisms potentially involved in AD etiology. At present, there is evidence for some major cellular/molecular pathways implicated in AD: (I) Aβ pathway progression, (II) inflammatory/immune response, (III) lipid homeostasis, (IV) oxidative stress response, (V) regulation of endocytosis and vesicle-mediated transport, (VI) regulation of cell cycle [30,31,32]. However, despite these progresses, a large part of the AD genetic background is still unidentified.

Although genetic factors appear to play a major role in the development of AD [13], there are several epigenetic modifications (e.g., DNA methylation, histone modifications, etc.) that likely contribute to its etiology and pathogenesis [20], including those caused by physical inactivity [33,34,35]. Physical inactivity is currently considered a pandemic, with one-third of all adults worldwide not meeting international physical activity (PA) recommendations [36,37]. In this regard, it has been estimated that approximately 13% of all cases of AD worldwide—and up to 21% and 31% in the US and Europe, respectively—are potentially due to physical inactivity [37]. By contrast, high PA levels have been reported as being beneficial for delaying the progression of the disease [35] and reducing its severity [33].

Different meta-analyses of randomized controlled trials (RCT) have reported significant benefits of exercise in patients with AD, particularly on cognitive function measures [38,39] and on the ability to perform activities of daily living [40]. Also, a meta-analysis of RCT and non-RCT showed significant benefits on cognitive function, activities of daily living, neuropsychiatric symptoms and physical function in those AD patients who were treated with exercise compared with those who were in control groups [41]. Despite the widely reported benefits of PA on AD, the underlying mechanisms remain still undefined. Given the multifactorial nature of AD [11], encompassing (epi)genetic and environmental factors, and its clinical and biological heterogeneity, using an holistic, integrative, and systems-level approach, at both experimental and computational level [9,42]—such as that provided by the systems biology paradigm—is required. Indeed, systems biology is a hypothesis-free, exploratory, and integrative paradigm aiming to explicate how complex interactions among different molecular entities—including DNA-protein, transcript-protein, miRNA-protein, protein-protein, protein-metabolite interaction networks—occur across structurally/functionally organized networks and systems, in both health and disease. Thus, systems biology helps better elucidate the cellular/molecular pathways and interactions involved in AD development and progression [43,44,45] and to explore the beneficial effects of protective strategies, such as PA [44].

In this context, this review summarizes the effect of PA on the different pathophysiological molecular pathways characterizing AD, assessed through the systems biology framework.

## 2. Physical Activity Effects on the Pathophysiological Molecular Pathways Associated with Alzheimer’s Disease

A systematic review of the literature—using the key terms “Alzheimer’s disease” AND “systems biology” in the PubMed database (search date: 12 October 2020)—was conducted to identify the pathophysiological molecular pathways associated with the development and/or progression of AD.

**Figure 1:**
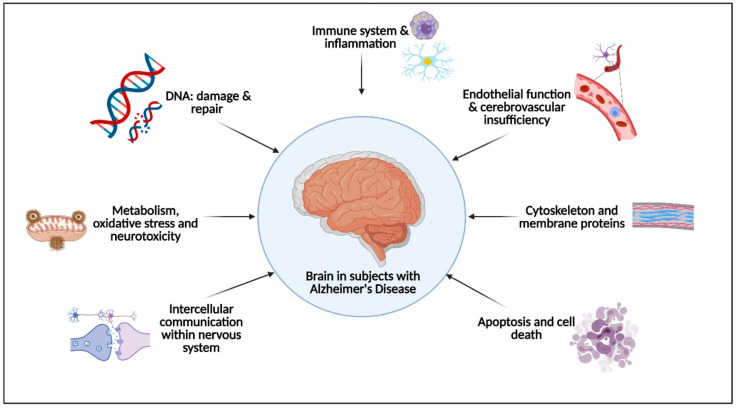
Alzheimer’s disease and related molecular pathways.

The following primary pathophysiological molecular pathways were identified by two independent authors (SLO and ASL) after the literature search for relevant articles (Figure 1, all images have been created using BioRender software [45]): (I) immune system and inflammation, (II) endothelial function and cerebrovascular insufficiency, (III) apoptosis and cell death, (IV) intercellular communication, (V) metabolism, oxidative stress and neurotoxicity, (VI) DNA damage and repair, (VII) cytoskeleton and membrane proteins, and (VIII) synaptic plasticity.

### 2.1. Immune System and Inflammation

#### 2.1.1. What Is It? How Does It Relate to AD?

**Figure 2:**
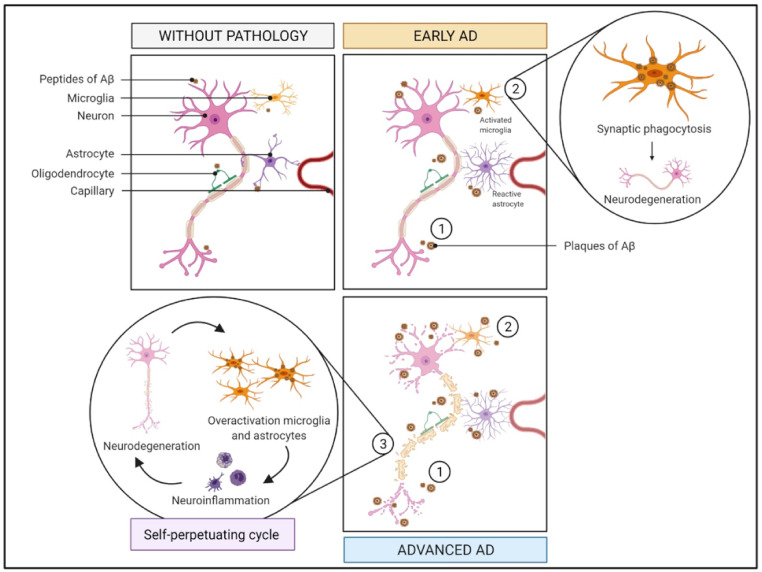
Immune system and inflammation pathway in AD. In AD, the accumulation of Aβ plaques (step 1) causes activation of microglia resulting in synaptic phagocytosis (step 2) and therefore neurodegeneration. This creates a self-perpetuating cycle (step 3) that increases neurodegeneration and induces a state of chronic neuroinflammation. Abbreviations: Aβ, amyloid beta; AD, Alzheimer’s Disease.

The response of the brain to toxic stimuli involves several cell types and molecular neuroinflammatory pathways (Figure 2). Glial cells are the resident immune cells of the brain and comprise two major subtypes—astrocytes and microglial cells—the acknowledged cell mediators of inflammatory pathomechanisms in AD.

Astrocytes are multifunctional cells that play a fundamental role in the development of neural circuits, as well as in synaptic pruning and metabolism [46,47]. They are involved in the spatial-temporal integration of several synaptic processes and modulate the synaptic strength and the neurotransmission. As a result, astrocytes participate in different physiological activities crucial for synaptic plasticity and cognitive activity [48].

Microglia are a category of mononuclear phagocytes/macrophages of hematopoietic origin that reside in the central nervous system (CNS) [49,50,51,52,53]. They represent around 12% of all brain cells [50] and are the primary component of the brain innate immune system [47,54]. They express various different receptors able to identify exogenous or endogenous CNS insults and initiate the immune response. In addition to their immune cell function, microglial cells protect the brain by inducing phagocytic clearance and providing trophic sustenance to maintain neuronal homeostasis and CNS plasticity [46,52,53,54,55]. Moreover, they have a central role in neuroinflammatory processes [51]. To provide these functions, microglia perform several physiological activities, including phagocytosis, cytokine production, complement activation, and generation of oxyradicals [56].

Both astrocytes and microglia respond to toxic stimuli in the brain by altering their gene expression, morphology, and secretion of paracrine factors [57,58], which have cascading effects on other types of cells, including neurons, the most affected cells in AD [57].

The loss and/or functional alteration of microglia may occur in response to neurodegeneration, thus contributing to the pathogenesis and progression of AD [54,55]. Microglia activation is crucial in the early stages of AD to clear extracellular Aβ plaques, but it can also lead to synaptic phagocytosis [49]. Such activation shows a high degree of heterogeneity, which can be generally categorized into two opposite activation phenotypes: “M1” and “M2”. Based on the specific phenotype activated, microglia can promote either cytotoxic or neuroprotective effects [23,25,59]: (i) M1, pro-inflammatory phenotype (classically activated), inducing a potent inflammatory response with the release of proinflammatory cytokines, such as interleukin-1β (IL-1β), IL-6, IL-12, and tumor necrosis factor-α (TNF-α) [23,25]; and (ii) M2, anti-inflammatory phenotype (alternatively activated), characterized by the production of anti-inflammatory cytokines, including IL-4, IL-10, or IL-13 [23]. Chronic and non-specific activation of microglia by Aβ plaques [47,56] is a common pathologic feature of AD that, in turn, can lead to an increase in the expression levels of proinflammatory mediators (e.g., IL-6, IL-1β and TNF-α) and neurotoxic molecules including nitric oxide [58,60,61]. When produced in large amounts, these cytotoxic molecules can damage brain cells, including neurons and other glial cells [60,61]. Injured neurons release damage-associated molecular patterns, which are recognized by microglia, triggering their activation. This creates a self-perpetuating cycle that exacerbates neurodegeneration and induces a state of chronic neuroinflammation [60] that promotes disease progression [58].

In addition to microglia, astrocytes are activated by tissue damage/injury or Aβ to secrete proinflammatory cytokines (e.g., IL-1, IL-6 or TNF-α), and massive astrogliosis has been reported in the brain of animal models of AD and of patients, with the accumulation of reactive astrocytes around Aβ plaques [22].

#### 2.1.2. How Does It Relate to Physical Activity?

There is extensive evidence on the effects of PA on the immune system, and how these effects are dependent on the nature and amount of the effort [62]. For example, long periods of intense PA can depress the immune system, whereas regular exercise at moderate intensity has beneficial effects [63]. Likewise, periods of acute exercise can induce oxidative stress and might act as a proinflammatory stimulus [21]; conversely, regular exercise can dampen the inflammatory response and might upregulate an endogenous anti-inflammatory response [21]. Indeed, exercise has been shown to inhibit microglial activation and improve AD pathogenesis in animal models and in patients by reducing the expression of inflammatory cytokines (e.g., IL-1β or TNF-α) [58,64]. In a rat model of AD, regular exercise facilitated the polarization of microglia from an M1 to an M2 state and improved cognitive function [27].

One of the mechanisms that can explain the anti-inflammatory effects of exercise is the production and release of myokines, particularly IL-6, by contracting muscles into the vascular system [21,64]. There is evidence that IL-6 has both proinflammatory and anti-inflammatory effects [64]. IL-6 can upregulate the expression of the anti-inflammatory cytokine IL-10 and the levels of the IL-1 inhibitor, IL-1 receptor antagonist [21,64], and it can also downregulate the expression of proinflammatory factors including TNF-α or IL-1β [21].

Exercise also enhances the levels of neurotrophic factors in tissues, such as brain-derived neurotrophic factor (BDNF) [58,63,65], which is primarily produced by brain microglia and astrocytes [64]. BDNF is known to reduce the levels of cytokines such as TNF-α, thereby alleviating symptoms of AD by reducing neuroinflammation [58]. Several studies suggest that physical exercise also favorably affects cellular markers associated with the disease, including the accumulation of Aβ plaques and the hyperphosphorylation of tau protein [60,66].

### 2.2. Endothelial Function and Cerebrovascular Insufficiency

#### 2.2.1. What Is It? How Does It Relate to AD?

**Figure 3:**
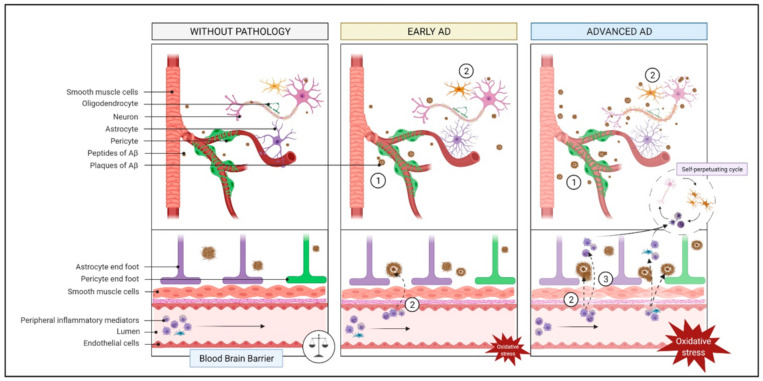
Endothelial function and cerebrovascular insufficiency pathway. In AD brains, the neuroinflammatory response causes an increase in the blood brain barrier (BBB) permeability. Also, the deposition of Aβ plaques in cerebral vessels (step 1) can augment BBB permeability (step 2), resulting in a release of peripheral inflammatory mediators on the brain, increasing the oxidative stress. This potentiates the self-perpetuating cycle of AD pathogenesis. Abbreviations: Aβ, amyloid beta; AD, Alzheimer’s Disease.

The neurovascular unit, primarily involving the cooperation among neurons, glial cells, endothelial cells, and smooth muscle cells [67,68,69,70,71,72,73], is responsible for the preservation of the brain homeostasis. Indeed, it closely monitors the brain chemical environment by maintaining an adequate cerebral blood flow (CBF) and modulating the transport of molecules across the blood-brain barrier (BBB) [67,68,70,71,72]. The BBB is a specialized structure that separates the CNS from peripheral tissues and serves to protect and regulate the brain internal milieu. Among its functions, the BBB limits the entry of peripheral inflammatory mediators (e.g., antibodies or cytokines) [70,72] that can blunt neurotransmission [70] (Figure 3).

Neuronal injury, neurodegeneration, or neuroinflammation typically induce disruptions of the BBB or the neurovascular unit. Loss of BBB integrity facilitates the entry of cytokines and immune cells into the CNS, which can in turn activate glial cells and cause secondary inflammation, thus exacerbating BBB damage and leading to its breakdown. BBB breakdown promotes the accumulation of neurotoxic material, the entry of microbial pathogens, dysfunctional BBB transport, inflammatory and immune responses leading to the generation of autoantibodies, red blood cell extravasation together with the release of neurotoxic free iron (Fe^2+^), which, in turn, produces reactive oxygen species (ROS) and oxidative stress [70,72,74]. The neuroinflammatory response in AD leads to damage endothelial cells and increase the BBB permeability [70], which is associated with reduced CBF and perturbed hemodynamic responses [71,74]. Decreased CBF in AD, as assessed by magnetic resonance imaging, has been reported in most brain regions, including the temporal, frontal and parietal lobes [28]. Furthermore, an increase in Aβ burden is associated with a reduction in CBF in the temporal lobes [30]. Pasha et al. observed that carotid stiffness, which reflects local cerebral arterial stiffness and can disrupt CBF regulation, was independently associated with the burden of brain Aβ in patients with mild-cognitive impairment [26]. In a similar context, brain amyloid angiopathy, which is caused by the deposition of Aβ in the cerebral vasculature, is associated with the degeneration of smooth muscle cells, pericytes and endothelial cells, triggering rupture of the BBB [72,74].

#### 2.2.2. How Does It Relate to Physical Activity?

Regular physical exercise reduces some risk factors (e.g., arterial hypertension) associated with impaired endothelial function [75,76], but it can improve endothelial function even without changes in blood pressure, lipid level, glucose tolerance, or body mass index [76]. The improvements in endothelial function induced by acute physical exercise at moderate intensity are driven by an increase in frictional forces (i.e., shear stress) exerted by blood flow in the endothelium of the vascular walls. Endothelial shear stress stimulates the production of vasodilatory substances such as nitric oxide [76,77,78] and increases the expression and activation of endothelial nitric oxide synthase [76], which promotes revascularization [36]. These improvements are also related to an increase in the abundance and mobilization of endothelial progenitor cells, which stimulate angiogenesis, promote vascular repair and inhibit atherosclerosis, in part mediated by nitric oxide [77,78].

Aerobic exercise training improves regional CBF in sedentary older men [11]. Likewise, 6 months of aerobic training was found to improve CBF in sedentary older adults with preclinical AD [32], and one year of aerobic exercise promoted an increase in CBF in individuals with mild cognitive impairment (MCI), which correlated with an increase in logical memory [31]. By contrast, no effects were observed in patients with mild-to-moderate AD after an aerobic training program of 16 weeks [79].

Physical exercise upregulates vascular endothelial growth factor (VEGF), which controls the angiogenic response to exercise [77,78,80,81], and maintains cerebrovascular integrity by sustaining blood flow and the supply of oxygen and nutrients to the brain [82]. Aerobic exercise can also improve endothelial function through the stimulation of peroxisome proliferator activated receptor-γ, which enhances the storage of fatty acids in adipose tissue, thereby reducing their levels in the bloodstream [24]. Aerobic exercise also induces the production of lactate, which activates the lactate receptor hydrocarboxylic receptor 1 to promote cerebral angiogenesis by enhancing VEGF levels in the brain [24].

The vascular fragility observed in AD affects some endothelial proteins such as endothelin-1, a vasoactive peptide that induces vasoconstriction and the proliferation of smooth muscle cells to increase vascular tone [75,78]. Physical exercise has been found to significantly decrease the plasma levels of endothelin-1 [78].

### 2.3. Apoptosis and Cell Death

#### 2.3.1. What Is It? How Does It Relate to AD?

**Figure 4:**
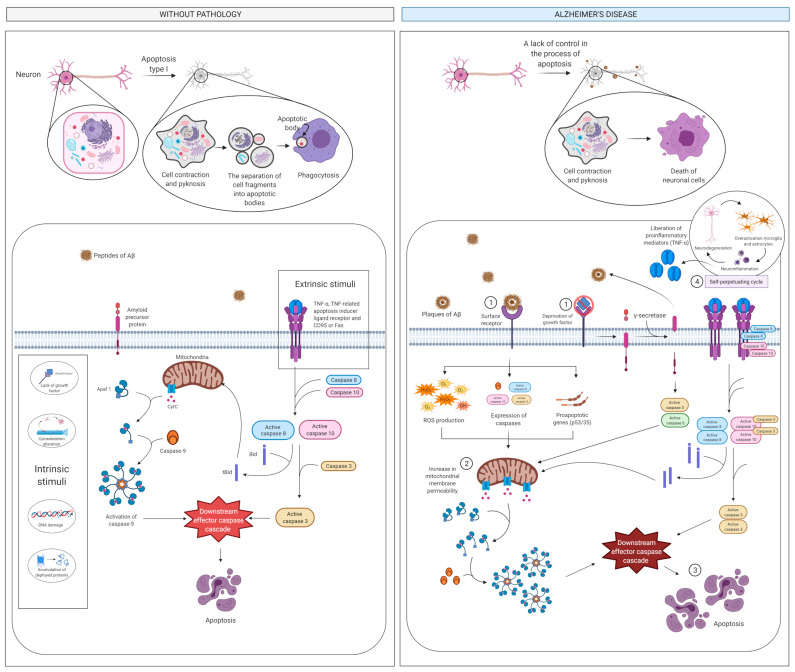
Apoptosis and cell death pathway. In the intrinsic pathway, the interaction of Aβ plaques with surface receptors of the neuron (step 1) results in ROS generation and expression of caspases and proapoptotic genes. In the extrinsic pathway Aβ produces a stimulus resulting in the activation of caspases. Both pathways produce an increase in mitochondrial permeability (step 2) and provokes the neuronal apoptosis (step 3) and therefore the activation of the self-perpetuating cycle (step 4). Abbreviations: Aβ, amyloid beta; Apaf 1, apoptotic protease-activating factor 1; CD95, cluster of differentiation 95; CytC, cytochrome C; H_2_O_2_, hydrogen peroxide; O_2_**^−^**, oxide; ROS, reactive oxygen species; t-Bid, truncated bid protein; TNF, tumor necrosis factor.

Neuronal apoptosis is considered as a key event in AD [83], although other processes are involved in the loss of neurons [84] (Figure 4). Aβ plaques interact with neuronal surface cell receptors and trigger the intrinsic apoptosis pathway by stimulating the production of ROS and the expression of caspases and pro-apoptotic genes, such as *p53*, and *BCL2*, which control mitochondrial membrane permeability. Aβ can also stimulate the extrinsic (non-mitochondrial) apoptotic pathway through its proinflammatory action, which stimulates astrocytes and microglia and triggers the liberation of proinflammatory mediators like TNF-α [83]. Also, deprivation of growth factors including nerve growth factor and BDNF induces abnormal amyloid precursor protein (APP) cleavage by β-site amyloid precursor protein cleaving enzyme-1 (BACE1 or β-secretase 1) that, in addition to γ-secretase, releases the toxic Aβ fragments and the soluble amino terminal N-APP fragment. N-APP, in turn, binds to the DR6 death receptor activating the caspases in both neuronal cell bodies (caspase-3) and axons (caspase-6). Specifically, caspase-6 can promote mitochondrial permeabilization leading to cytochrome c release and the stimulation of executioner caspases that regulate the beginning of the “execution phase” of the apoptosis. [83].

#### 2.3.2. How Does It Relate to Physical Activity?

Physical exercise modulates the concentration of cytokines, hormones, growth factors and the oxidative state. In addition, exercise affects energy balance through the mobilization and metabolism of substrates such as carbohydrates or free fatty acids. All of these factors are known to mediate and even slow death or prolong cell survival [85].

As mentioned earlier, physical exercise reduces the levels of cytokines (e.g., TNF-α) released during microglia activation [58]. Moreover, PA beneficially influences cellular markers associated with AD, reducing the accumulation of Aβ plaques [34,60,66]. Also, exercise is well recognized to elevate the levels of neurotrophic factors (e.g., BDNF and VEGF), promoting neurogenesis, neuroprotection and cell survival [34,65,82].

Finally, aerobic physical exercise increases the levels of telomere-stabilizing proteins, which protect against cellular senescence, and decreases the abundance of apoptotic regulators [86].

### 2.4. Intercellular Communication

#### 2.4.1. What Is It? How Does It Relate to AD?

Changes in protein homeostasis at the presynaptic level, leading to synaptic pathology, are associated with many neurological disorders, including AD [87]. Loss of neurotrophic support, as can occur when levels of BDNF are low, is linked to reduced neuronal survival and synaptic plasticity [65]. The first manifestations of memory loss in AD are related to synaptic dysfunction and loss [57,87]. In addition, several studies show that build-up of APP may reduce the plasticity of nearby neurons [88], and that deposition of Aβ in synaptic spaces may cause a reduction in synaptic density [57,87,88].

##### The Cholinergic System

**Figure 5:**
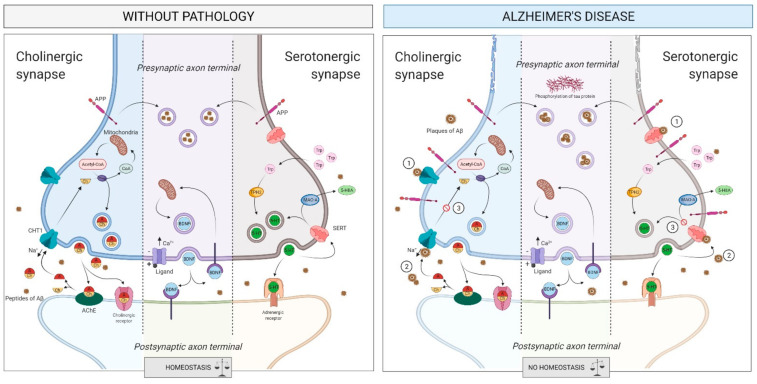
Intercellular communication within nervous system pathway. In AD, the accumulation of Aβ in the surface receptors (step 1) and the synaptic spaces (step 2) causes a reduction in neurotransmitter levels, impeding the recapture of these (step 3) and increasing the phosphorylation of tau protein and the creation of Aβ plaques inside the axon. Abbreviations: 5-HIIA, 5-hydroxy indoleacetic acid; 5-HT, 5-hydroxytryptamine or serotonin; Aβ, amyloid beta; Acetyl-CoA, acetyl coenzyme-A; Ach, acetylcholine; AChE, acetylcholinesterase; APP, amyloid protein precursor; BDNF, brain-derived neurotrophic factor; Ca^2+^, calcium ion; CHT1, high-affinity choline transporter 1; CoA, coenzyme-A; MAO-A, monoamine oxidase A; Na^+^, sodium ion; TPH2, tryptophan hydroxylase 2; Trp, tryptophan; SERT, serotonin transporter.

The main AD pathophysiological hallmarks, i.e., brain Aβ deposition and tau protein hyperphosphorylation, are complexly related to the loss of cholinergic neurons and cortical cholinergic innervations [89,90,91]. In particular, loss of cholinergic neurons detected in the basal forebrain (BF) is a consistent finding in AD patients. The study of the pattern of cortical projections from the BF cholinergic nuclei showed that cholinergic axons are predominantly sent to the hippocampus and the cerebral cortex [92,93]. It also seems that AD may be associated with a vicious circle of cholinergic depletion, which intensifies the production and neurotoxicity of Aβ which, in turn, increases the deficit of the cholinergic axonal projections and, therefore, the hyperphosphorylation of tau protein [90] (Figure 5). In this regard, post-mortem studies reported that the depletion of the cortical cholinergic innervations is associated with (and, most likely, caused by) the NFTs in the nucleus basalis of Meynert (NBM) within the BF [92,94]. The NBM is a major source of cortical cholinergic innervation subject to severe neurodegeneration in AD [95,96]; the cholinergic loss is due to the degeneration of NBM cholinergic neurons and of their axons projected to the cerebral cortex. The BF cholinergic neurons are among the cell bodies most inclined to neurofibrillary degeneration and NFTs creation [92].

Besides NFTs, Aβ deposition has also been described in the BF. Such amyloid deposits, whose distribution is mainly circumscribed to the NBM, are attributable to congophilic angiopathy associated with amyloid plaques and NFTs. Furthermore, the density of Aβ deposits correlates with cell depletion in the NBM [97]. However, since studies exploring correlations between cholinergic denervation and the density of Aβ deposits in AD-affected human brains present conflicting results, further investigations are needed [98].

Another common finding in AD is the depletion of cortical cholinergic markers, including a significant reduction of the levels of cortical acetylcholine (ACh) and the activities of choline acetyltransferase (ChAT) and acetylcholine esterase (AChE) enzymes (i.e., the ACh synthesizing and degrading enzymes, respectively) [99,100,101]. The marked reduction in ChAT and AChE enzymatic activities in AD is associated with both a significant increase in the mean plaque count and the degree of cognitive decay [101,102,103].

##### The Serotonergic System

Serotonergic alterations seem to be intimately related to behavioral aspects of AD, such as depression, aggressive behavior, and psychosis [104,105]. Some of the serotonin modulators induce changes in the expression or processing of APP, therefore affecting the amyloidogenic pathway [106]; moreover, serotonin transporter (SERT) expression is decreased in patients with AD, thus appearing a reliable neural marker associated with memory mechanisms [104]. Furthermore, decreased blood concentrations of serotonin have a potential role in cognitive impairment during AD progression [107,108]. Lastly, plasma concentrations of tryptophan—the serotonin precursor—appear to be decreased in early AD patients, thus explaining alterations in the serotonin pathway [109,110].

Recently, plasma concentrations of metabolites from both cholinergic and serotonergic pathways have been found to be altered in MCI due to AD (MCI-AD) participants compared to healthy control individuals and linked to cognitive impairment and neurodegeneration. This has allowed the development of a model based on specific metabolites (betaine, cytidine, uridine, choline, acetylcholine, serotonin, and tryptophan) that has a potential usefulness in AD diagnosis [111].

#### 2.4.2. How Does It Relate to Physical Activity?

By stimulating the production of neurotrophic factors such as BDNF, PA promotes neurogenesis [34,35] in the hippocampus and synaptic plasticity [35], increasing the number of synapses and dendritic receptors, and stimulating neuronal growth and survival [82]. BDNF is a neurotrophin that signals through tropomyosin kinase B [80] to modulate neurogenesis, synaptogenesis and dendritogenesis [65,80]. Acute or regular physical exercise increases the levels of BDNF both in serum [65] and in the hippocampus [80,81], which improves cognitive function. The levels of other neurotrophins are increased with exercise, such as nerve growth factor, which is the main protector of cholinergic neurons in the telencephalon, a brain region essential for learning and memory that is damaged in AD [80].

Physical exercise influences the release of neurotransmitters related to cognitive function, increasing their levels in the CNS, such as ACh, noradrenaline [65] or serotonin [65,81]. This increase and the cooperation between neurotransmitter and neurotrophins, may promote beneficial, long-lasting neural adaptations [65].

### 2.5. Metabolims, Oxidative Stress and Neurotoxicity

#### 2.5.1. What Is It? How Does It Relate to AD?

**Figure 6:**
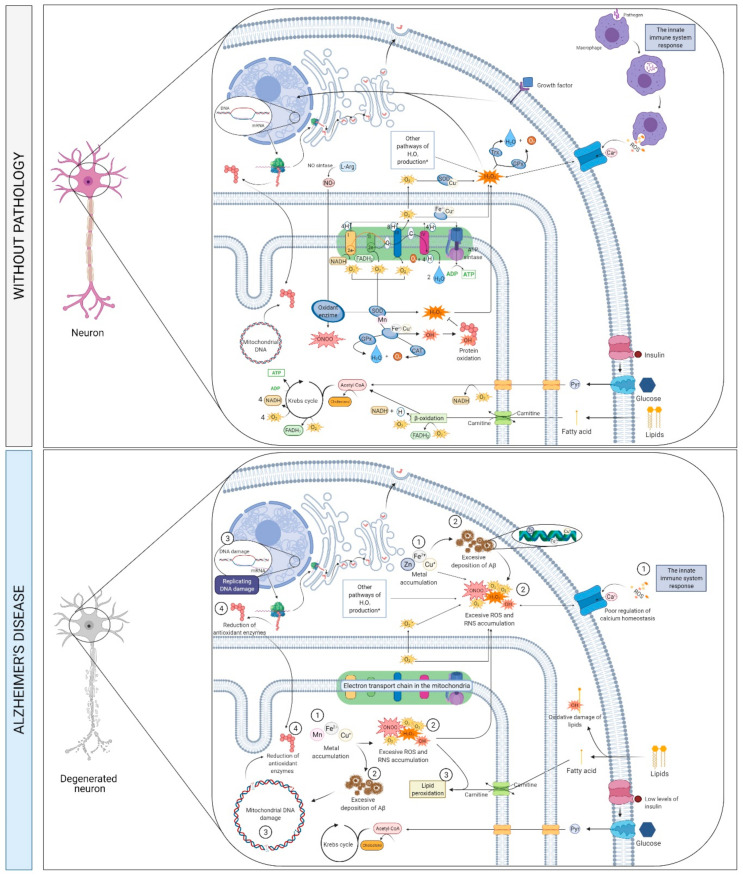
Metabolism, oxidative stress and neurotoxicity pathway. Abnormal accumulation of Aβ plaques, loss of mitochondrial function, increased production of ROS, metal dyshomeostasis, and diminished antioxidant protection mechanisms are all occurring during the progression of AD pathophysiology. Mitochondria participate in multiple cellular/molecular activities, such as energy metabolism, ATP synthesis, Ca^2+^ signaling, and iron (among other metals) homeostasis. Therefore, neuronal viability is greatly dependent on mitochondrial activity. Thus, mitochondrial deficiencies are frequently detected in neurodegenerative diseases, including AD. Since mitochondria are the primary source of ROS, any abnormality in the proper function of the electron transport chain results in damage of a number of biomolecules (i.e., proteins, lipids, nucleic acids). In patients with AD, Aβ accumulation is associated with impairment of mitochondrial activity, decreased oxidative phosphorylation, and ROS generation. As a result, a reduction of energy supplies is observed. In AD brains, ROS levels are significantly more elevated than in healthy brains. During the generation of Aβ plaques, several ROS species are produced; particularly, H_2_O_2_ is one of the most relevant ROS species. The ROS/H_2_O_2_ production further triggers production and aggregation of Aβ, which, in turn, can lead to ROS/H_2_O_2_ generation. Bidirectional interactions between ROS and Ca^2+^ signaling pathways are typical: ROS is able to modulate cellular Ca^2+^ signaling, which in turn is crucial for ROS production. Hence, increased levels of Ca^2+^ activate the enzymes responsible for the creation of ROS and free radicals. The ROS-Ca^2+^ interplay participates in several pathophysiological conditions, for instance AD, Parkinson’s disease, inflammatory diseases, and cancer. AD patients also show altered homeostasis of metals, including iron, copper, or zinc, that damages the cell redox system and promotes oxidative load as well as an increased deposition of extracellular Aβ plaques. Oxidative load induces the production of elevated amounts of end-products of lipid peroxidation, various different oxidized proteins, and oxidative alterations in both nuclear and mitochondrial DNA. Abbreviations: Aβ, amyloid beta; AD, Alzheimer’s disease; Ca^2+^, calcium; H_2_O_2_, hydrogen peroxide; ROS, reactive oxygen species.

Mitochondrial dysfunction and the abnormal accumulation of transition metals can lead to excessive ROS generation and Aβ plaques deposition [83,112,113,114] (Figure 6), thereby triggering oxidative stress and neurodegeneration [83,112], and eventual hyperphosphorylation and polymerization of tau protein [83,115,116]. Mitochondrial dysfunction may result in sub-optimal regulation of Ca^2+^ homeostasis and an increase in the production of ROS, both of which are related to neurotoxicity [112]. The interaction of copper, iron or zinc with Aβ plaques, as well as metal-mediated oxidative stress, is associated with the pathogenesis of AD [116,117,118], and can occur because of a failure in the metabolism of metals, i.e., metal dyshomeostasis [119].

In AD brains, the levels of peroxisome proliferator-activated receptor gamma co-activator 1alpha (PGC-1α), which is an essential protein that modulates mitochondrial biogenesis and regulates oxidative stress, are decreased [120,121], so it can lead to mitochondrial dysfunction and to a dysregulated expression of uncoupling protein 2 (UCP2), which is a direct regulator of ROS formation expressed in the hypothalamus [120,122]. PGC-1α can inhibit Aβ generation through a reduction in β-secretase 1 (BACE1) expression, transcription, and a decrease in BACE1 promoter activity [123]. Thus, a reduced expression of PGC-1α in the hippocampus is correlated with the progression of amyloid neuropathy in AD patients [124]. Panes et al. considered that PGC-1α “sequestered” in the cytosol could be the non-return point for the neuron on the Aβ toxicity [125]. Low levels of PGC-1α are also associated with abnormal brain insulin signaling [126].

Abnormal insulin metabolism is also important in this pathway. Insulin resistance syndrome occurs when tissues do not respond to insulin [127], which leads to hyperinsulinemia [127,128] and a decrease in insulin transport through the BBB. Reduced insulin signaling in the brain is associated with increased phosphorylation of tau protein and levels of Aβ [127,128] and with oxidative stress, thus affecting systems that mediate neuronal plasticity and cognitive functions [128]. A failure of insulin metabolism as well as increased oxidative stress can lead to vascular dysfunction, which aggravates the symptoms of AD [113,127].

Lipids represent a relevant component of brain structure and function. They can be categorized into sphingolipids, glycerophospholipids, and cholesterol [129]. The cerebral fatty acid (FA) composition is highly distinctive and consists of long-chain polyunsaturated FA (LC-PUFA)—representing around 50% of neuronal membrane—mainly eicosapentaenoic acid (EPA), docosahexaenoic acid (DHA), arachidonic acid (AA), and docosapentaenoic acid (DPA). Both EPA and DHA originate from alpha-linolenic acid (ALA), an omega-3 FA [129,130] whereas AA and DPA originate from linolenic acid (LA), an omega-6 FA [131]. Omega-3 and 6 FA are the most common LC-PUFA in the brain [132]. These LC-PUFA are integrated into membrane phospholipids and have a major role in the structural integrity and activity of cell membranes.

During aging, the metabolism of FA and lipids is altered, as reported by decreased levels of omega-3 FA and intensified lipid peroxidation [132]. Because omega-3 FA show antioxidant properties, a deficiency of these FA in the diet may facilitate neurodegeneration [133]. Given the mounting indication of the association between AD and dysregulated FA metabolism, the levels of FA and lipids are expected to be candidate biomarkers of AD [134,135].

Moreover, recent GWAS have detected associations between genes implicated in cerebral lipid homeostasis—*APOE*, clusterin (*CLU*), sortilin-related receptor 1 (*SORL1*), and ATP-binding cassette sub-family A member 7 (*ABCA7*)—and the risk of developing AD [136,137].

Abnormalities in FA and lipid metabolism can be responsible for dysfunctional brain networks related to AD pathophysiology. In particular, dyshomeostasis of lipid metabolism is associated with atypical APP processing, altered neuronal receptor-mediated signaling pathways, increased/persistent inflammation, oxidative stress, perturbed BBB integrity and function, myelin synthesis disruption, and mitochondrial dysfunction. Ultimately, these pathophysiological processes result in neurodegeneration [138].

##### APP Proteolytic Processing

The link between APP proteolytic processing and lipid homeostasis is increasingly explored. APP is created in the endoplasmic reticulum of neurons and conveyed to the cell surface via the trans-Golgi network; its intracellular trafficking is accurately modulated [139]. Different pathways exist concerning the proteolytic processing of APP, the amyloidogenic and non-amyloidogenic pathways [140]. Particularly, the latter is initiated after APP is internalized in the endosomes and cleaved by BACE1, placed on the endosomal membrane [138].

Interestingly, BACE1 showed inhibited enzymatic activity in environments with reduced or absent cholesterol, indicating that cholesterol and, more in general, the lipid arrangement of the intracellular environment is a potential key factor regulating BACE1 entrance to APP endosomes [138]. Additional analyses indicate that both lipid configuration homeostasis and lipid oxidation state are crucial to APP processing modulation [141,142].

In the amyloidogenic pathway of APP processing, the occurrence of saturated and oxidized FA induces the disruption of the cell membrane architecture and facilitates BACE1 activation. Under increased concentrations of oxidized lipids, soluble APPα (sAPPα) species levels are reduced, while soluble APPβ (sAPPβ) levels are more elevated [143]. Furthermore, lipid mediators of inflammation communicate with APP processing through the O-linked-N-acetylglucosaminylation (O-GlcNAcylation) [144]. Ultimately, oxidized or inflammatory lipids might promote the shift of APP proteolytic processing from the non-amyloidogenic to amyloidogenic pathway [138].

Both APP and BACE1 are strictly linked to lipid rafts, which are dynamic microdomains containing sphingolipids, cholesterol, and phospholipids (particularly phosphatidylcholine). Such lipids are essential to vesicle trafficking and intracellular transport [145]. Indeed, lipid rafts encompass resident proteins (lipid raft-associated proteins) [146], and work as multimolecular platforms where protein complexes are interconnected to modulate signal transduction pathways [147]. During the lipid dyshomeostasis process, lipid rafts exhibit modifications in their molecular structure [148]. Alterations in lipid rafts’ organization and architecture might affect their physicochemical features modifying the local microenvironment. These changes might induce a reorganization of the lipid raft-associated proteins [149], ultimately expected to trigger neurodegeneration [148].

An increase in the neuronal requirements of cholesterol—needed, for instance, to remodel membranes—induces the expression and secretion of apolipoprotein E. APOE4 isoform expression is decreased when the levels of cerebral cholesterol increase, thus affecting the process of dendritic, axonal, and synaptic regeneration [150]. Elevated cholesterol levels are related to an increase in the production of Aβ and also affect the activity of β- and γ-secretases in the amyloidogenic pathway [113,151].

Individuals who carry the *APOE ε4* allele are more likely to develop AD because the APOE4 protein can increase the deposition of Aβ, whereas the *APOE ε2* allele appears to have a protective effect [152,153]. However, it seems to be a risk factor for neurodegeneration associated with tau protein [153].

#### 2.5.2. How Does It Relate to Physical Activity?

Paradoxically, as mentioned earlier, although acute physical exercise induces an increase in oxidative stress [36,76], regular physical exercise is related to an improved redox status [34,35,80,114] as well as an improvement in clearing and activity of Aβ degrading enzymes [35,114]. Physical exercise reduces oxidative stress related to vascular dysfunction, improving the release of nitric oxide and vasodilation [34,154]. Furthermore, higher levels of exercise are associated with reduced levels of brain Aβ plaques in patients with AD [43].

PGC-1α appears to mediate many cellular effects of exercise by its interaction with fibronectin type III domain-containing protein 5 (FNDC5), the precursor of a myokine called irisin [122,155]. It has been demonstrated that physical exercise can increase the expression of PGC-1α and FNDC5 in animal models [122,156]. Also, it has been proved that exercise can increase UCP2 levels in the hippocampus in rats and its antioxidant effects [122]. Physical training enhances the plasma levels of irisin, increasing the energy consumption of adipose tissue and inducing BDNF expression [156]. The exercise-induced upregulation of PGC-1α improves both lipid metabolism and insulin sensitivity [157].

Physical exercise is also related to metabolic conditions such as lipid dysfunction and insulin resistance [154], reducing the blood levels of cholesterol and insulin [35]. Regular exercise increases high-density lipoprotein (HDL) cholesterol levels while maintaining those of low-density lipoprotein (LDL) cholesterol and triglycerides [158]. Endurance training increases reliance on lipids as an energy substrate, inducing systemic lipid-lowering effects resulting in skeletal muscle lipid metabolism towards increased oxidation neutral lipid storage and turn-over [159]. During exercise, the FA transport by carnitine through the mitochondrial membrane is increased, regulating FA oxidation [160]. Also, physical activity decreases lipid peroxidation [161], and short and long-term endurance training increases antioxidant enzyme activities in trained subjects [162]. However, it has been shown that acute periods of high-intensity endurance training in previously untrained subjects increases lipid peroxidation [162].

Additionally, both aerobic and resistance training improve insulin sensitivity [163], and acute exercise increases the muscle uptake of glucose [164].

### 2.6. DNA: Damage and Repair

#### 2.6.1. What Is It? How Does it Relate to AD?

**Figure 7:**
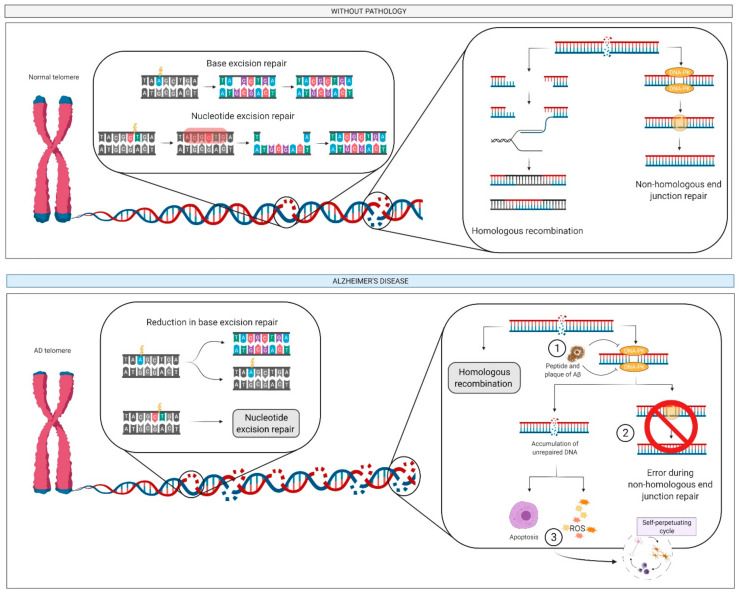
DNA: damage and repair pathways. The deposition of Aβ peptides (step 1) can difficult the non-homologous end junction repair (step 2) leading to the accumulation of numerous lesions of DNA, resulting in apoptosis and ROS generation (step 3). This can activate the self-perpetuating cycle of AD. Abbreviation: Aβ, amyloid beta; AD, Alzheimer’s disease; DNA, deoxyribonucleic acid; ROS, reactive oxygen species.

Defective DNA repair is observed in several neurodegenerative diseases including AD [165], and is associated with elevated levels of DNA double-strand breaks and reduced levels of DNA repair proteins. In addition, Aβ peptides can inhibit DNA-dependent protein kinase, which limits DNA repair by the non-homologous end-joining pathway [165] (Figure 7). The accumulation of DNA lesions in the neuronal genome as a result of this failure might direct cells to premature senescence or apoptosis, ultimately leading to the loss of synapses, chronic inflammation or protein aggregation, and neurodegeneration [165].

#### 2.6.2. How Does It Relate to Physical Activity?

Exhausting physical activity without appropriate recovery periods can increase inflammation and ROS generation, resulting in DNA damage [166]. This damage is, however, transitory and directly related to intensity and recovery periods, and hence repeated sessions of exhaustive physical exercise with inadequate recovery periods can reduce the efficiency of the antioxidant system and DNA repair [166].

The association between physical exercise and the release of BDNF has been confirmed in numerous studies [58,63,65,167]. Interestingly, BDNF can protect neurons from death through a mechanism that involves the regulation of DNA damage repair [167], and so the greater the release of BDNF, the better the DNA repair and the lower the risk of neurodegeneration.

### 2.7. Cytoskeleton and Membrane Proteins

#### 2.7.1. What Is It? How Does It Relate to AD?

**Figure 8:**
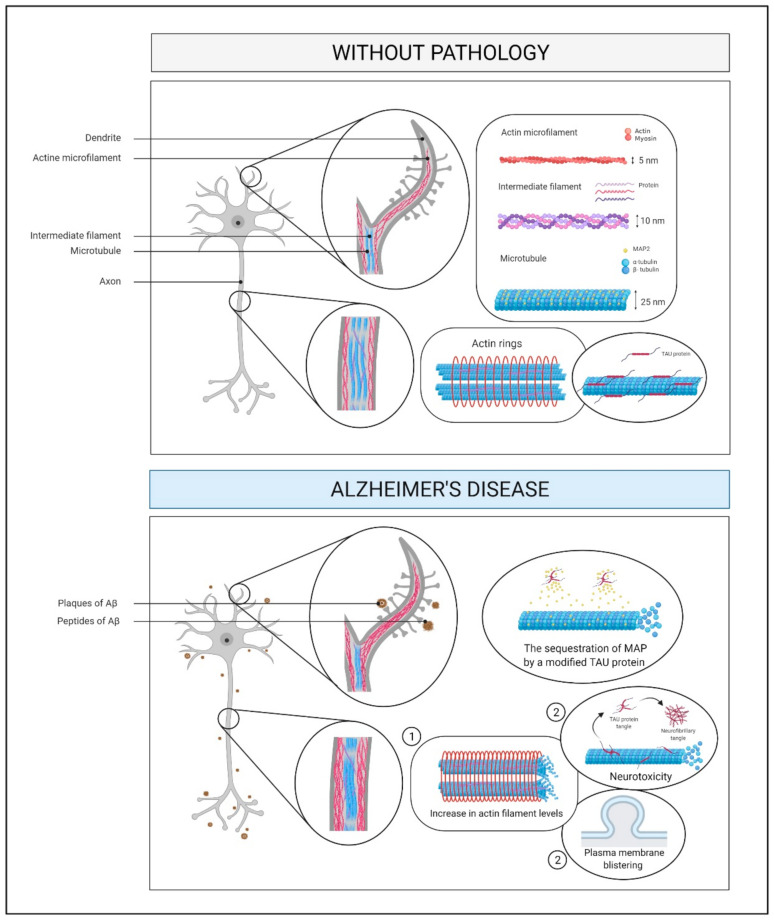
Cytoskeleton and membrane proteins pathway. The neurotoxicity induced by tau protein is associated with an increase in actin filament levels (step 1) and cytoskeleton remodeling, causing plasma membrane blistering and more neurotoxicity (step 2). Also, a reduction in the dynamism of microtubules is caused by the sequestration of MAP2 by a modified tau protein. Abbreviations: Aβ, amyloid beta; MAP2, microtubule-associated protein 2; nm, nanometers.

Neurodegenerative diseases may be related to abnormalities in the structure of the cytoskeleton [168]. For example, tau-induced neurotoxicity is associated with an increase in the levels of actin filaments and cytoskeleton remodeling, which can cause plasma membrane blistering [169] (Figure 8). The dynamism of microtubules in AD is affected by disease mechanisms such as the alteration of the assembly of microtubules through the sequestration of microtubule-associated protein by modified tau [170]. Microtubule dynamics are essential for the regulation of dendritic morphology and synaptic plasticity, and so disturbances can cause synaptic loss [170].

#### 2.7.2. How Does It Relate to Physical Activity?

The elevated oxidative stress after strenuous PA can lead to oxidative modifications of cytoskeleton proteins [8]. Also, physical exercise increases the levels of several proteins related to cytoskeletal function and neuronal development, such as β-tubulin [11]. In addition, BDNF/tropomyosin kinase B receptor signaling activates the release of a specific neuronal protein (Shank) that controls the actin cytoskeleton in dendritic spines and their regression [12]. The levels of microtubule-associated protein 2 in the hippocampus are also elevated with exercise [12], resulting in improved axonal regeneration.

In a preclinical rat model, moderate physical exercise was found to change the expression of synaptic proteins and cytoskeletal neurofilaments, which may trigger plasticity in regions of the brain that are related to motor function [9].

### 2.8. Synaptic Plasticity

#### 2.8.1. What Is It? How Does It Relate to AD?

Synaptic plasticity is a biological process by which different patterns of synaptic activity (Figure 5) cause modifications of synaptic strength which are thought to underlie learning and memory [171,172]. Alteration of these processes contributes to a wide range of neurological and cognitive disorders [173] including AD [174], even though the molecular mechanisms remain poorly understood [175]. In animal models of AD, impaired synaptic function is an early event leading to decline in memory processing long before the occurrence of amyloid plaque burden and neuronal cell death [176]. Of note, long-term potentiation (LTP) is often used to evaluate the phenotype associated with the different AD mouse models and has been instrumental in the validation of pharmacological [177,178] or non-pharmacological approaches, including physical exercise [179].

#### 2.8.2. How Does It Relate to Physical Activity?

Physical exercise modulates a number of signaling pathways involved in synaptic plasticity even though the exact mechanisms are still unclear. One of the first evidences linking exercise to synaptic plasticity and cognitive function in AD model was provided by Garcia-Mesa and colleagues [180]. Using in vivo electrophysiological recordings, authors investigated synaptic transmission and plasticity in 3xTg-AD mice that were exposed to 6-month physical exercise. In this condition, wheel running treatment was able to slightly rescue the decline of LTP at the CA1-medial prefrontal cortex synapse.

More compelling results were obtained by Liu and colleagues [181], which showed that treadmill exercise reversed LTP impairment and enhanced learning and memory in the APP/PS1 transgenic model. Surprisingly, this protective effect was still evident in APP/PS1 mice at 17 months of age, a phase associated with considerable Aβ plaque deposition [182].

Another group has extensively investigated the effects of treadmill exercise on learning and memory and synaptic plasticity in a rat model of AD [183,184,185]. In this model, results consistently suggest a neuroprotective effect induced by exercise. Specifically, Aβ-treated rats manifested deficiency in learning and short-term memory and decreased early LTP (E-LTP) in area CA1. Notably, a regimen of moderate treadmill fully reversed learning and memory and LTP impairments [184]. From a mechanistic point of view, in amyloid-infused rats exercise normalized specific plasticity-related signaling pathways such as Ca^2+^/calmodulin-dependent protein kinase II (CaMKII), calcineurin (protein phosphatase 2B [PP2B]), and BDNF. These data suggests that by re-establishing a correct kinase-phosphatase balance, treadmill exercise is able to prevent dentate gyrus synaptic alterations typically associated with AD [183]. In a subsequent study, the same authors investigated the effect of physical exercise on spatial long-term memory and the late-phase LTP (L-LTP) in the dentate gyrus and CA1 subregions. L-LTP decline was reversed in exercised Aβ-treated rats, and this was paralleled by increase in the levels of phosphorylated (p)-CREB, Ca^2+^/calmodulin-dependent protein kinase IV (CaMKIV), and BDNF [185].

Beside functional plasticity, also structural plasticity is affected by training. Accordingly, training promoted an enhanced dendrite ramification, spine generation and plasticity in the dentate gyrus of a sporadic model [186] and a transgenic model [187] of AD, which are dependent on CaMKII activation.

Among the mediators linking physical exercise to synaptic plasticity and memory, FNDC5/irisin has been shown to play a key role [188]. Indeed, FNDC5/irisin levels are reduced in hippocampi and cerebrospinal fluid of individuals with AD, as well as in mouse models of AD. This may account for the impairment of LTP and novel object recognition memory observed in AD mice. On the other hand, promoting brain levels of FNDC5/irisin via physical exercise was able to rescue LTP and memory in this model [188], possibly representing a valid therapeutic approach to protect synapse integrity and prevent cognitive decline in AD.

## 3. Investigating the Link between Alzheimer’s Disease and Physical Exercise through Neuroimaging Technologies

During the last few years, numerous imaging methods, modalities, and analysis algorithms have been used as response markers for therapeutic strategies applied to AD-related dementia. In this context, it is well-known that physical exercise can significantly support pharmacological therapy in AD. For example, previous studies have evidenced significant effect of physical training on brain atrophy and, particularly, on hippocampal volume, a typical hallmark of AD [189]. More in detail, structural magnetic resonance imaging (MRI) allows the quantification of brain atrophy, which is a significant feature of neurodegeneration, and it has become a key tool to investigate disease progression and to monitor therapeutic efficacy. While in clinical practice evaluation of temporal atrophy can be performed through qualitative (observer-dependent) or semi-quantitative methods, nowadays automated segmentations techniques are preferred for an independent and quantitative volumetric calculation of hippocampal volume. In addition, automated image analysis methods permit the calculation of atrophy distribution in the entire brain, through either measurements of regions of interest, maps based on grey matter density or cortical thickness, and volume measurements [190].

Using such techniques, several studies found a correlation between increased physical fitness and bilateral hippocampal volume [191], while also examining specific subregions of the hippocampus; in particular, it was found that the positive effects of physical fitness were most evident in the anterior regions [192,193]. Also, a longitudinal analysis found that an increase in cardiorespiratory (CR) fitness over two years was associated with a reduction in medial temporal atrophy in AD patients [194]. Beyond grey matter atrophy, the study of white matter (WM) through diffusion weighted imaging has provided a wealth of additional information about AD pathology; in particular, multiple diffusion tensor imaging (DTI) studies have shown that not only gray matter, but also WM, is affected in its microstructural integrity in AD patients, demonstrating the high sensitivity of DTI in detecting early microscopic WM degeneration. DTI has demonstrated that WM is involved in cognitive impairment, as changes in the connections of the hippocampus [195], posterior cingulum [196], thalamus [197], and regions in the posterior WM were found. In this context, there have been reports of cross-sectional association between DTI-derived measures in WM and cardiovascular fitness, and DTI may provide useful information about WM microstructure before and after aerobic exercise [198]. Also, it has been reported that higher levels of cardiorespiratory fitness were associated with greater WM integrity in the right inferior fronto-occipital fasciculus (IFOF), possibly indicating that a higher exercise regimen may preserve integrity in WM tracts that have not already been compromised by the neurodegenerative process [199]. MR-perfusion weighted imaging (PWI), and a recently introduced technique called arterial spin labelling (ASL), allows the noninvasive estimation of the CBF. ASL is commonly preferred to other perfusion methods in both young and older adults because it labels protons in arterial blood using radiofrequency pulses, without resorting to exogenous injection of paramagnetic contrast agents. Global and regional CBF decrease has been demonstrated in AD patients in specific areas of the brain, which included the cingulum, precuneus, parietal lobes, and lower frontal regions. Interestingly, reduced CBF has been linked to disease severity in AD patients [200] in possible association with reduced tissue metabolic demand [201]. In this context, one group employed MRI with pulsed arterial spin labeling (PASL) sequences to assess CBF changes in AD patients undergoing therapeutic exercise and concluded that there may not be an effect of exercise on CBF in individuals with mild to moderate AD [79].

Finally, the benefits of exercise have also been demonstrated using functional magnetic resonance imaging (fMRI) techniques. In particular, fMRI imaging of the brain during cognitive exercises has been able to demonstrate functional improvements in cognitive and cortical networks which were associated exercise or fitness. In this context, promising results from a number of randomized clinical trials in a population of young adults have shown increased connectivity and cortical activity associated with aerobic exercise compared to controls [202,203]. Also, as multicentric, multimodal, and multi-scale databases are growing, the need for a holistic integration of neuroimaging, biohumoral, and genetic and neuropsychological data in order to augment the predictive and discriminatory power of additional therapeutic boosters like physical exercise is becoming increasingly evident. In this context, novel image processing technologies based on deep learning, which are flexible enough to make such predictions while including neuroimaging as well as non-neuroimaging data, are currently being developed [204].

## 4. Conclusions

Several molecular pathways appear to be involved in the development and/or progression of AD, notably a pro-inflammatory oxidative state, impaired endothelial function, enhanced apoptosis and neuronal death, impaired intercellular communication, DNA damage, and abnormalities in the cytoskeleton.

In turn, accumulating evidence suggests that PA might be an effective strategy for counteracting these processes and delaying the development and progression of AD.

## Data Availability

Not applicable.

## Abbreviations

Aβ, amyloid beta peptide; Aβ_1–42_, the 42 amino acid-long Aβ peptide; AA, arachidonic acid; ABCA7, ATP-binding cassette sub-family A member 7; ACh, acetylcholine; AChE, acetylcholine esterase; AD, Alzheimer’s disease; ALA, alpha-linolenic acid; APOE, apolipoprotein E; APP, amyloid precursor protein; ASL, arterial spin labelling; BACE1, β-site amyloid precursor protein cleaving enzyme-1; BBB, blood-brain barrier; BDNF, brain-derived neurotrophic factor; BF, basal forebrain; CaMKII, Ca^2+^/calmodulin-dependent protein kinase II; CaMKIV, Ca^2+^/calmodulin-dependent protein kinase IV; CBF, cerebral blood flow; ChAT, choline acetyltransferase; CLU, clusterin; CNS, central nervous system; CR, cardiorespiratory; DHA, docosahexaenoic acid; DPA, docosapentaenoic acid; DTI, diffusion tensor imaging; E-LTP, early long-term potentiation; EOAD, early-onset AD; EPA, eicosapentaenoic acid; FA, fatty acids; fMRI, functional magnetic resonance imaging; FNDC5, fibronectin type III domain-containing protein 5; GWAS, genome-wide association studies; IFOF, inferior fronto-occipital fasciculus; LOAD, late-onset AD; IL, interleukin; LA, linolenic acid; LC-PUFA, long-chain polyunsaturated fatty acids; LTP, long-term potentiation; L-LTP, late-phase long-term potentiation; MCI, mild cognitive impairment; MRI, magnetic resonance imaging; NBM, nucleus basalis of Meynert; NFTs, neurofibrillary tangles; NGS, next-generation sequencing; O-GlcNAcylation, O-linked-N-acetylglucosaminylation; PA, physical activity; PASL, pulsed arterial spin labeling; PSEN1, presenilin 1; PSEN2, presenilin 2; PP2B, protein phosphatase 2B; PWI, perfusion weighted imaging; ROS, reactive oxygen species; SERT, serotonin transporter; SORL1, sortilin-related receptor 1; sAPPα, soluble sAPPα; sAPPβ, soluble APPβ; TNF-α, tumor necrosis factor-α; VEGF, vascular endothelial growth factor; WM, white matter.
